# Melt electrowriting of PLA, PCL, and composite PLA/PCL scaffolds for tissue engineering application

**DOI:** 10.1038/s41598-022-24275-6

**Published:** 2022-11-19

**Authors:** Mohammad Shahverdi, Saeed Seifi, Ali Akbari, Kaivan Mohammadi, Amir Shamloo, Mohammad Reza Movahhedy

**Affiliations:** 1 Advanced Manufacturing Laboratory, School of Mechanical Engineering, Sharif University of Technology, Azadi Ave., Tehran, Iran; 2 Nano BioTechnology Laboratory, School of Mechanical Engineering, Sharif University of Technology, Tehran, Iran

**Keywords:** Biological techniques, Engineering

## Abstract

Fabrication of well-ordered and bio-mimetic scaffolds is one of the most important research lines in tissue engineering. Different techniques have been utilized to achieve this goal, however, each method has its own disadvantages. Recently, melt electrowriting (MEW) as a technique for fabrication of well-organized scaffolds has attracted the researchers’ attention due to simultaneous use of principles of additive manufacturing and electrohydrodynamic phenomena. In previous research studies, polycaprolactone (PCL) has been mostly used in MEW process. PCL is a biocompatible polymer with characteristics that make it easy to fabricate well-arranged structures using MEW device. However, the mechanical properties of PCL are not favorable for applications like bone tissue engineering. Furthermore, it is of vital importance to demonstrate the capability of MEW technique for processing a broad range of polymers. To address aforementioned problems, in this study, three ten-layered box-structured well-ordered scaffolds, including neat PLA, neat PCL, and PLA/PCL composite are fabricated using an MEW device. Printing of the composite PLA/PCL scaffold using the MEW device is conducted in this study for the first time. The MEW device used in this study is a commercial fused deposition modeling (FDM) 3D printer which with some changes in its setup and configuration becomes prepared for being used as an MEW device. Since in most of previous studies, a setup has been designed and built for MEW process, the use of the FDM device can be considered as one of the novelties of this research. The printing parameters are adjusted in a way that scaffolds with nearly equal pore sizes in the range of 140 µm to 150 µm are fabricated. However, PCL fibers are mostly narrower (diameters in the range of 5 µm to 15 µm) than PLA fibers with diameters between 15 and 25 µm. Unlike the MEW process of PCL, accurate positioning of PLA fibers is difficult which can be due to higher viscosity of PLA melt compared to PCL melt. The printed composite PLA/PCL scaffold possesses a well-ordered box structure with improved mechanical properties and cell-scaffold interactions compared to both neat PLA and PCL scaffolds. Besides, the composite scaffold exhibits a higher swelling ratio than the neat PCL scaffold which can be related to the presence of less hydrophobic PLA fibers. This scaffold demonstrates an anisotropic behavior during uniaxial tensile test in which its Young’s modulus, ultimate tensile stress, and strain to failure all depend on the direction of the applied tensile force. This anisotropy makes the composite PLA/PCL scaffold an exciting candidate for applications in heart tissue engineering. The results of in-vitro cell viability test using L929 mouse murine fibroblast and human umbilical vein endothelial (HUVEC) cells demonstrate that all of the printed scaffolds are biocompatible. In particular, the composite scaffold presents the highest cell viability value among the fabricated scaffolds. All in all, the composite PLA/PCL scaffold shows that it can be a promising substitution for neat PCL scaffold used in previous MEW studies.

## Introduction

3 Dimensional (3D) polymeric scaffolds as a platform for cell culture and growth have a significant role in tissue engineering. They can be used as a promising alternative for autografts, allografts, and xenografts^1^. Until now, researchers have shown the application of the scaffolds in cutaneous^2^, musculoskeletal^3^, liver^4^, cardiovascular^5^, and nervous^6^ tissue engineering. To regenerate injured tissue in the best way, scaffolds must have essential characteristics like biocompatibility and biodegradability. Furthermore, they should be able to mimic the mechanical properties of the tissue in the implantation site. The architecture of the scaffold and its fabrication technique are other important factors that should be considered about these structures^7^.

There are several techniques for fabrication of scaffolds. Techniques like gas foaming^8^, freeze drying^9^, solvent casting/particulate leaching^10^, thermally induced phase separation^11^, and electrospinning^12^ are the conventional techniques used for scaffold fabrication^13,14^. Among these techniques, researchers’ attention has considerably been drawn to electrospinning technique due to very high surface-to-volume ratio of electrospun scaffolds as well as the high throughput of materials in this technique^15^. However, electrospun scaffolds inherently suffer from the lack of controlled deposition of fibers. Moreover, because of random deposition of fibers, the obtained microarchitecture and particularly small pore size associated with electrospun scaffolds are not suitable for applications such as osteogenesis (which needs pore size equal to ~ 100 µm^16^) and vascularization (the optimum pore size for vascularization is ~ 400 µm^17^).

Recently, biofabrication techniques rooted in additive manufacturing (AM) principles have been the focus of numerous studies in the field of tissue engineering and specifically scaffold fabrication. One of the most important advantages of these methods over traditional techniques is the complete control they offer over the architecture of the scaffold^15^. Melt electrowriting (MEW) that was first introduced by Brown et al.^18^ is a novel AM technique for scaffold fabrication. MEW combines the two well-established techniques of fused deposition modeling (FDM) and electrospinning. In MEW, a polymer melt heated in a syringe flows from the tip of a metallic nozzle to a substrate/collector while a very large electric potential difference between the nozzle and the grounded substrate stabilizes the melt flow during its flight toward the collector^19,20^. Usually, a back-pressure created using air is utilized to deliver the polymer melt to the nozzle head. A g-code is used to program the motion of the substrate or nozzle head in x and y directions with a desired velocity. MEW is capable of fabrication of well-ordered scaffolds with micron to sub-micron fibers, interconnected pores, and high porosity.

Until now, MEW of a variety of polymers (including polycaprolactone (PCL)^17,21–24^, polypropylene^25^, poly(2-ethyl-2-oxazoline)^26^, poly(2-ethyl-2-oxazine)^27^, and poly(vinylidene difluoride)^28^) and copolymers (such as poly(lactic-*co*-glycolic acid)^29^, poly(urea-siloxane)^30^, poly(_L_-lactide-*co*-acryloyl carbonate)^31^, and poly(_L_-lactide-*co*-ε-caprolactone)^32^) has been successfully demonstrated. The gold standard polymer used in most studies for MEW process is PCL^20^. PCL is a hydrophobic polyester and an ideal choice for MEW process because of its low melting temperature, fast solidification, low rate of degradation, and biocompatibility^20^. Although the compatibility of the mentioned polymers with MEW process has been shown, there is still a large number of biocompatible and biodegradable polymers with applications in tissue engineering and scaffold fabrication that are left to be melt electrowritten, one of which is poly(lactic acid) (PLA)). PLA is a synthetic polyester which its cytocompatibility and biodegradability have made it a desired candidate for different applications in biomedical and tissue engineering^33^. Several studies have used PLA scaffolds fabricated using different techniques like electrospinning for tissue engineering applications^5,34,35^. Besides, MEW of this polymer has been investigated in several studies^36–39^. Zhang et al.^36^ combined an electrohydrodynamic jet printing device and an FDM 3D printer to fabricate PLA scaffolds. What they actually conducted was MEW of PLA. However, they did not call their setup an MEW device. After them, one research group published three papers on printing PLA scaffolds using MEW process^37–39^. In the first paper published by this group, they mentioned that they fabricated PLA scaffolds using MEW process for the first time. The printed PLA scaffolds had a fiber diameter and pore size of 40 µm and 200 µm, respectively^37^. In the next two studies, they tried to solve problems associated with melt electrowritten PLA scaffolds. In one of the papers, they developed a method based on robust alkaline treatment to add bioactivity to the melt electrowritten PLA scaffolds^38^. In the last paper, they used melt electrowritten PLA fibers merged with gelatin/genipin/bioglass hydrogel to develop a composite scaffold. The composite scaffold demonstrated improved mechanical properties and provided a better environment for bone tissue growth compared to melt electrowritten PLA scaffold^39^.

Fabrication of composite scaffolds using two or more types of polymers is a promising technique to weaken the deteriorating effects caused by the inherent drawbacks of the single polymers. One of the well-known examples is the composite scaffold made of PCL and PLA polymers with altered properties compared to neat PLA and PCL^40–44^. Although PLA has great advantages like high elastic modulus and excellent biocompatibility and biodegradability that make it a promising choice for bone tissue engineering applications, its brittleness has severely limited its use. One way to improve this drawback is to blend PLA with PCL which is a ductile polymer with low glass transition temperature^40,42^. It has been reported that the composite polymer obtained by blending PLA and PCL has a lower modulus and yield stress compared to neat PLA. Furthermore, adding PCL to PLA improved ductility and flexibility of PLA as well as increased its elongation and impact toughness^43,44^. The composite polymer was observed to have a better thermal degradation behavior than that of PLA^41^. In a study, using a dual-jet electrospinning device, PCL and PLA solutions were simultaneously electrospun onto a grounded collector to fabricate nanofibrous mats^45^. It was observed that the rate of weight loss of the mats which was an indication of hydrolytic degradation was higher at a pH of 10^45^. The enhanced properties of the composite PLA/PCL polymer have led to the fabrication of composite PLA/PCL scaffold with tissue engineering applications^46,47^. Furthermore, Patricio et al.^46^ showed that compared to the scaffold made of neat PCL, the adhesion and proliferation of cells was increased for the PLA/PCL scaffold.

In this study, MEW process is used to fabricate PLA, PCL, and PLA/PCL scaffolds. Unlike conventional MEW devices used in most of previous studies, here, a commercial FDM device is utilized for MEW of PLA fibers. To make it compatible with the MEW process, modifications in the setup of the FDM 3D printer are carried out. Use of a conventional FDM 3D printer as an MEW device is one of the novelties of this study. Also, fabrication of the composite PLA/PCL scaffold using MEW process is done for the first time. All scaffolds are made of 10 layers of microfibers placed in the box structure of the scaffold. The composite PLA/PCL scaffold has an alternating pattern in which PLA and PCL microfibers are laid in consecutive layers. Multi-material scaffolds made using traditional techniques like electrospinning or solvent casting/particulate leaching suffer from random distribution of different materials^48,49^. This is a serious drawback which makes the behavior of the cells cultured on the scaffold unpredictable. MEW with the described operating mechanism resolves this issue and helps fabricating a more tissue-like structure. The fabricated scaffolds are characterized and compared in terms of fiber morphology, pore structure, mechanical properties, interaction with water, and biodegradation rate. In vitro experiments including cell culture and viability are conducted with L929 mouse murine fibroblast and human umbilical vein endothelial (HUVEC) cells to demonstrate the potential of the scaffolds as promising candidates for tissue engineering applications.

## Materials and methods

### Materials

PCL and PLA filaments with medical grade are acquired from Advance Biomedical Technology Inc. (Taiwan). Tetrazolium dye for the MTT assay is purchased from Sigma Aldrich (USA). Glutaraldehyde (GA) is provided from TAAB Company. L929 mouse murine fibroblast and human umbilical vein endothelial (HUVEC) cells were purchased from GenIran Company.

### MEW device and its operating parameters

**Figure 1:**
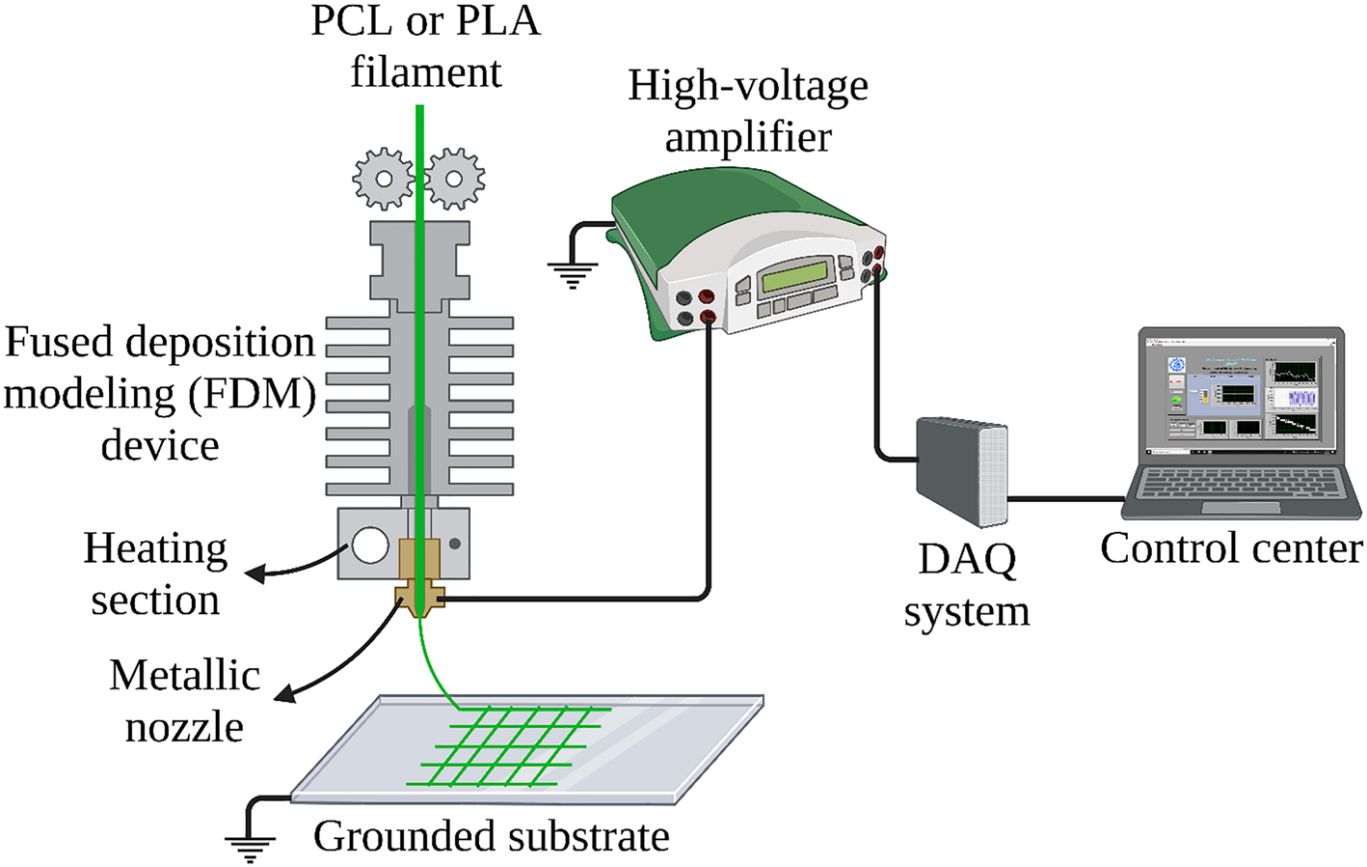
Schematic of the experimental setup (drawn on the BioRender website (https://biorender.com) by the authors).

**Figure 2:**
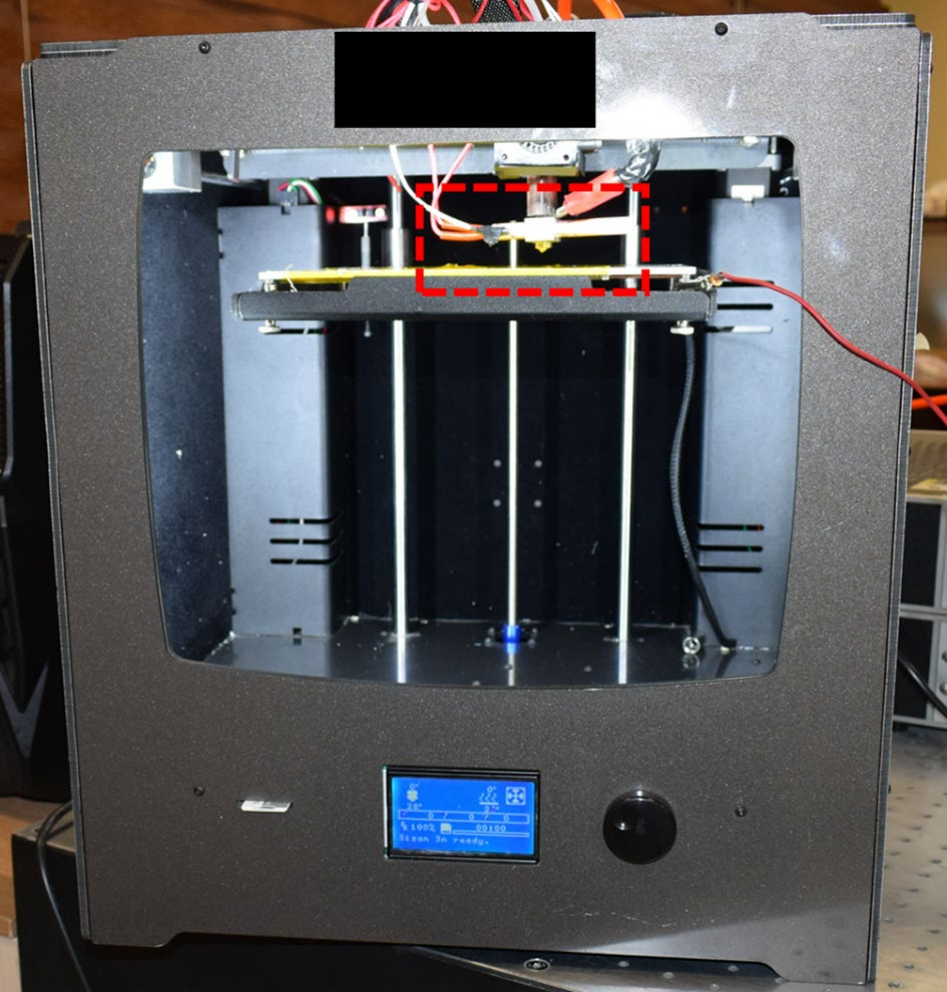
FDM 3D printer used in this study as the MEW device.

The schematic of the experimental setup is represented in Fig. 1. MEW device used in this study is in fact a commercial FDM 3D printer (Fig. 2) that has been previously used for fabrication of typical 3D printed parts. To 3D print a part, a filament of PLA or acrylonitrile butadiene styrene (ABS) with a certain diameter of 1.75 mm is fed to a metallic nozzle with an opening diameter of 200 µm through a gear system which includes two gears. Using a gear system instead of applying a large back-pressure to a polymer melt in a heated reservoir is one of the main differences of this system with conventional MEW devices reported in most of the previous studies. When a gear system is used, the flow rate/feed rate can be controlled directly by setting the rotational velocity of the gears and the properties of the polymer melt cannot influence the feed rate/flow rate. However, this is not the case for conventional MEW devices. For these systems, what the device operator can control is the back-pressure. The flow rate/feed rate, in this case, is calculated from principles of fluid mechanics and depends on the rheological properties of the polymer melt (like viscosity). Therefore, with a constant back-pressure, by varying the polymer, the feed rate/flow rate will also change.

In the FDM device used in this study, the motion of the filament starts from the gear system. After passing through the two gears, the filament enters a plastic tube and its motion is guided by this tube. At the end of the tube, the filament reaches the cooled end section of the FDM device. The next section which the filament should pass through after the cooled end box is the heating section. The temperature of the filament in the heating section rises to a value equal to or larger than the melting temperature of the filament. The temperature of the heating section is controlled using a temperature sensor. The part of the filament that is inside the heating section melts and the molten polymer flows from the nozzle tip and deposits on the surface of a heated substrate/collector. The temperature of the collector is set to a much lower value than the melting temperature of the filament. In this way, when the molten filament places on the substrate, it solidifies rapidly. It should be noted that this temperature is high enough to cause the deposited filament to fuse to its upper and lower layers.

The continuous feeding of the polymer to the heating box by the gear system causes a sustained polymer melt to flow from the nozzle tip. One of the problems associated with the gear system is that since a stepper motor is utilized to rotate the gears, when low feed rates are needed (which is the case for MEW process) and the stepper motor should rotate with very small angular velocities, it may rotate in discontinuous steps and with time lags and therefore, the rotational motion of the gears is badly affected and the filament will not be fed to the nozzle tip continuously. One way to overcome this problem is to add a gearbox system with a gear ratio of 10 to be able to deliver very small feed rates persistently.

In this study, the uniform diameter of the printed fibers which is evidenced by the SEM images from the structure of the melt electrowritten scaffolds shows that, in the feed rates used for the MEW process, the problem of the disrupted feed rate does not exist.

A problem here is related to those parts of the filament which are near the heating section. As the polymer inside the heating section melts, the temperature of the filament before the heating box increases and this may cause that these parts of the filament lose their solid form. This, in turn, may lead to difficulties in the guidance of the filament motion in the sections before the heating part. To overcome this problem, a fan is provided in the cooled end which lowers the temperature of the filament and does not allow the filament to lose its solid form.

In the FDM device of this study, the nozzle head and substrate are able to move in x–y and z directions, respectively. During 3D fabrication of a part, using a g-code program prepared in Cura software, all parameters including the feed rate, x and y coordinates and velocity of the nozzle head, temperature of the nozzle head and substrate, and z coordinate of the substrate are determined and controlled continuously.

To find the value of the suitable feed rate, first, the values of nozzle head velocity, nozzle diameter, and layer height are entered in Cura software and then the software calculates the value of the feed rate based on the three mentioned parameters. The value obtained for the feed rate can be changed by varying a percentage value in Cura software. A 100 percentage (default mode) is associated with feed rate values that are suitable for the fabrication of conventional parts by the FDM device. To reach the optimized value of the feed rate for the MEW process, different values (lower than 100) for the percentage are tested. Fabrication and evaluation of scaffolds with different percentages help to find the optimum percentage and feed rate.

**Figure 3:**
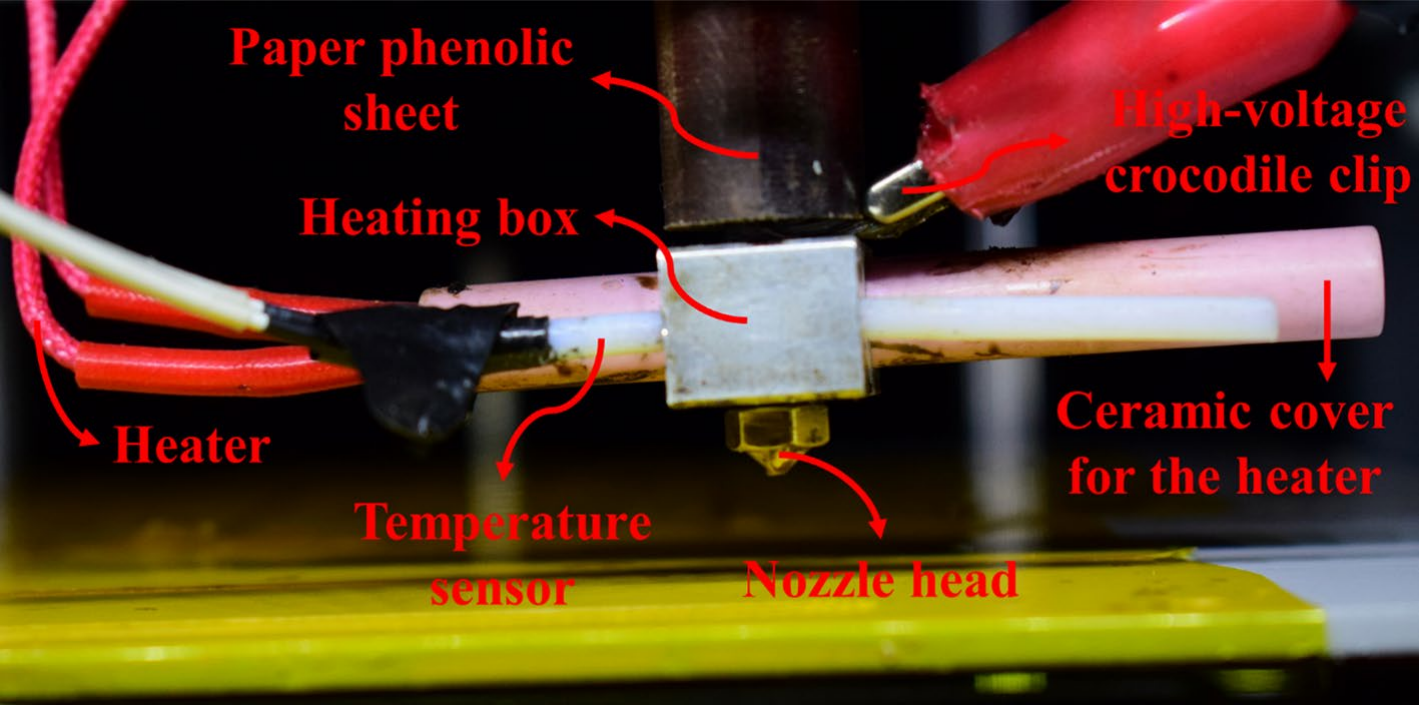
Print head and heating section of the modified FDM device and the configuration used to confine the high voltage to nozzle head and heating box.

To convert the FDM device into an MEW printer, changes should be carried out. To apply a high voltage to the metallic nozzle, it is connected to a high-voltage (HV) amplifier (Trek HV Amplifier, model: 10/10-2, State College, PA, 16803, USA). The HV amplifier is able to generate an HV in the range of 0 to 10 kV. When the HV is applied, the electrical connection between the metallic nozzle and other parts of the FDM printer may cause a great damage to the FDM device and for this reason, the nozzle should be electrically isolated from other parts of the device. In Fig. 3, the nozzle head and heating section as well as the configuration used to localize the high voltage is shown. It should be noted that Fig. 3 is actually the magnified view of the dashed red box in Fig. 2. As it can be seen from Fig. 3, right before the nozzle, the heating section is placed and therefore, electrical separation should be carried out between these two sections. However, in the FDM device used in this study, the relative configuration of the box of the heating section and the metallic nozzle makes it impossible to isolate these two parts from each other. So, to solve the problem between these parts, for the heating section, a ceramic heater (which is insulator) and a temperature sensor with a thin insulating cover are used. The next thing to do is to electrically confine the high voltage to the heating section and to prevent the high voltage from causing damage to other parts of the FDM 3D printer. This is performed by placing a tube made of paper phenolic sheet before the heating box. The PLA or PCL filament has to pass through this tube to enter the heating part of the FDM printer. As the last step, the substrate and the metallic nozzle should be isolated. The substrate itself is metallic and therefore, is conductive. The existence of the strong electric field which is created when the HV is applied to the nozzle as well as the small distance between the nozzle tip and the substrate may cause an electrical discharge between these two parts which so harmful and dangerous for both of the FDM device and its operator. Here, Kapton sheets are placed on the metallic substrate to separate it electrically from the nozzle. Placing these insulating sheets badly affects the quality of MEW process (because electrical charges accumulate on the surface of insulating Kapton sheets and this deteriorates the electric field between the metallic nozzle and the substrate). However, this is the best option to prevent electrical discharge.

The HV amplifier is connected to a personal computer (PC) through a data acquisition (DAQ) system (multifunction I/O device, National Instruments (NI), M/N: USB-6001, P/N: 154424D-03L, Austin, TX, USA). The DAQ system communicates with the PC using a LabVIEW program. It receives the signal containing the value of the direct current (DC) voltage and sends it to the HV amplifier and then, the amplifier applies the desired voltage to the metallic nozzle. The last thing that has to be done is to connect the substrate to the ground. During the MEW process, by applying the HV to the nozzle, a strong electric field is created between the nozzle and the substrate. The electric field stabilizes the FDM jet even when the feed rate is low and this allows the fabrication of micron scale fibers from PLA or PCL filaments.

### Scanning electron microscopy (SEM)

Images taken using the SEM device can be used for the characterization of scaffold structure and microfibers morphology as well as calculation of pore size and porosity of the scaffolds. Moreover, adhesion of cells onto the surface of the microfibers can be investigated utilizing these photographs. In this study, a field emission SEM (FE-SEM) device (MIRA 3, TESCAN, Brno, Czech Republic) is used to take images from different parts of the scaffolds at various magnifications. Samples should be electrically conductive for SEM and FE-SEM, and hence, before placing the samples in the FE-SEM device, the surface of the polymeric scaffolds (that are non-conductive) is coated with a thin layer of gold using a sputter coating device (DSR1, Nanostructured Coatings Co., Tehran, Iran).

To calculate the pore size, fiber diameter, and porosity of the scaffolds, after taking the images, ImageJ software is used. For the pore size, from each sample scaffold 20 pores are randomly selected and after calculation of the size of each pore, the average pore size is reported. For the case of the fiber diameter, the same procedure conducted for the pore size is utilized with a difference that here the diameter of 20 random microfibers is calculated and then, the average value is stated. Eventually, for calculation of the porosity, each image is turned into a black and white picture with the microfibers and pores as the black and white parts, respectively. Then, ImageJ software calculates the surface area of the whole scaffold as well as the black and white parts, and based on the calculated values, the porosity is computed and reported.

### Tensile test

As mentioned in the introduction section, the mechanical properties of the scaffolds are very important and they should be similar to those of the natural tissue that the scaffold is going to be replaced with. In this context, the uniaxial tensile test finds its place as one the most necessary characterization tests for a scaffold.

In this research, uniaxial tensile test is performed using a universal testing machine (Hounsfield-H10KS, USA). Before test, each sample scaffold is cut to 1 cm × 5 cm strips. One of the strips, then, is placed between two grippers of the machine and is elongated until failure. The distance between the grippers is constant in all tests and equal to 3 cm. A 100 N load cell is used to elongate the strip with an elongation rate of 0.5 mm/min. From the tensile tests, the values of stress and strain at the yield, ultimate (peak), and failure points could be calculated and determined. Moreover, the slope of the stress-vs-strain curve gives the value of the Young’s modulus.

### Atomic force microscopy (AFM)

Surface topography and roughness are characterized using an AFM device (Ara-AFM, Ara Research, Iran) with HQ:NSC15 cantilever (tip radius: ⁓ 8 nm, typical resonance frequency: 325 kHz, typical spring constant: 40 N/m) in non-contact mode. From each scaffold one microfiber was detached and its surface properties was evaluated in a 500 nm × 500 nm window (the window has a resolution of 256 pixels × 256 pixels). Using the Gwyddion software, the root mean square (RMS) value of the surface roughness (R_q_) is calculated for all points of the window and then, its average and standard deviation values are reported in the form of average ± standard deviation.

### Water contact angle and swelling

Using a static contact angle meter device (Contact C1, Iran) and deionized water, the degree of hydrophobicity/hydrophilicity (surface wettability) of the scaffolds is determined.

For swelling test, first, one of the microfibers in the scaffold is marked and the diameter of that microfiber (D_0_) is measured using a handheld digital microscope (Dino-Lite Digital Microscope, AnMo Electronics Co., Taiwan) and DinoCapture 2.0 software (Dino-Lite Digital Microscope, AnMo Electronics Co., Taiwan). Then, the whole scaffold is immersed in water. After one day of immersion in water, the diameter of the marked microfiber (D_1_) is calculated again using the same digital microscope and software. Based on the two measured diameters, the percent swelling ratio (SR) is obtained from the below equation:

1 $$SR (\%)=\frac{{D}_{1}-{D}_{0}}{{D}_{0}}\times 100.$$

### In vitro experiments

#### Cell culture and MTT assay test

One of the most important evaluation tests for the performance of a scaffold is its ability in keeping cells that are in contact with the scaffold alive^50^. To investigate this ability, MTT assay is used^51^. In this technique, oxidoreductase enzymes in alive cells reduce soluble MTT with a chemical formula of 3-(4,5-dimethylthiazol-2-yl)-2,5-diphenyltetrazolium bromide and yellow color to purple insoluble formazan. The purple formazan is first dissolved in dimethyl sulfoxide (DMSO) and then, extracted from the living cells. The absorbance of the extracted purple solution is evaluated using an ELISA plate reader in the wavelength of 500–600 nm. Solutions with higher concentrations of formazan have a deeper purple color and therefore, absorb higher wavelengths.

In this study, L929 mouse murine fibroblast and HUVEC cells are used to evaluate the potential of the scaffolds in keeping cells alive^52,53^. Before culturing the cells, the scaffolds are sterilized using ethanol and ultra-violet (UV) light. First, they are soaked in 70% ethanol for 2 h and then, under cell culture hood, their both sides are exposed to UV light for 1 h. To clean the scaffolds from ethanol, they are thoroughly washed with sterilized phosphate-buffered saline (PBS) (Merck co., USA) for three times and each time for 20 min. To be sure about the scaffolds being sterilized, they are dipped in the cell culture media inside an incubator. The temperature and relative humidity of the incubator are set to 37 °C and 80%, respectively, and its atmosphere contains 5% CO_2_ gas. After staying at this condition for 24 h, if no contamination occurs in the media of cell culturing, scaffolds are ready for MTT assay test.

Cell culturing is conducted in the same way for both L929 and HUVEC cells. The first step for this is pouring the cell culture media on the scaffolds. Using a Neubauer lam and trypan blue dye, the number of defrosted cells that are passaged and grown is measured. Using this method, it is possible to distinguish living cells from dead ones. In the next step, 50,000 cells are placed on each of the scaffolds that have been previously put in the wells of a microplate. Finally, each scaffold is placed in cell culture media containing 1 ml of the Dulbecco’s Modified Eagle Medium (DMEM) (Idea Zist Recombinant Co., and 10% fetal bovine serum (FBS) (Idea Zist Recombinant Co., Iran), and is kept in the cell culture incubator.

Acridine orange/ethidium bromide (AO/EB) is utilized to observe the morphology of L929 and HUVEC cells cultured on scaffolds on day 4 via fluorescence microscope (Labomed, U.S.). In addition, MTT assay test is conducted in days 1 and 4 of cell culture. In each of these days, first, a volume of 550 µl is extracted from the cell culture media of each scaffold and 50 µl of the solution of yellow color MTT in PBS with a concentration of 5 mg/ml is added to it. The samples are maintained for 3 to 4 h in the cell culture incubator. Next, the cells are taken out of the incubator and 500 µl DMSO is added into the wells of the microplate to dissolve the purple formazan. The microplate containing the samples is placed for 20 min in a shaking incubator with a temperature and rotational velocity of 37 °C and 120 rpm, respectively, while the microplate is covered with an aluminum foil to prevent exposition of the samples to light. Eventually, 100 µl of the media around each sample is taken and then, injected into each well of another microplate. A microplate reader device (ELx800™, BioTek Instruments Inc., USA) is used to measure the concentration of the purple color at a wavelength of 570 nm. This is the procedure used to evaluate the cell viability in each of days 1 and 4. A controlled test in which cells are cultured in a media without any scaffold is also carried out. The controlled test is used to calculate the percent cell viability based on the below relationship:

2 $$Cell \, viability \left(\%\right)=\frac{number \, of \, alive \, cells \, in \, the \, presence \, of \, a \, scaffold}{number \,of\, alive\, cells\, in\, the\, control\, test}\times 100.$$

#### Cell adhesion

Adhesion of cells to the surface of the scaffold significantly affects their growth and proliferation. To investigate cell adhesion, SEM is used to record images from the cells attached to the surface of the scaffolds.

In this study, adhesion of L929 and HUVEC cells to the surface of the scaffolds is explored. The first step is to culture cells in a similar procedure to that described in the “Cell culture and MTT assay test” subsection. Cell adhesion is studied in days 1 and 4 of culturing. In each of these days, first, cell culture media is discharged and then, 1 ml of 2.5% solution of glutaraldehyde (Merck co., USA) is added to each well to stabilize the cells. The samples are kept for 3 h in darkness with glutaraldehyde. After that, glutaraldehyde is thrown away and each scaffold is washed three times with PBS. Afterwards, the samples are respectively dipped in 50%, 60%, 70%, 80%, 90%, and 100% ethanol, each for 15 min and the scaffolds are placed in a refrigerator to become completely dried. After this step, the scaffolds are ready for Au sputter coating and SEM imaging.

### Statistical analysis

Substantial variations among the results of different samples are investigated statistically. Here, the method used for studying this is the one-way analysis of variance (ANOVA) together with Tukey’s honestly significant difference (HSD) post hoc test. GraphPad Prism 9 (GraphPad Software, CA, USA) is utilized to statistically analyze the results. Values of p that are smaller than 0.05 indicate statistical significance.

## Results and discussion

### MEW process

There are several parameters affecting the morphology and other properties of the melt electrowritten scaffolds. These parameters can be related to the properties of the polymer (including electrical conductivity, melt viscosity, and dielectric constant) as well as to the settings of the MEW device itself (such as applied voltage, feed rate, nozzle head velocity, and stand-off height). Also, it is important whether the substrate/collector is made of an electrical conductor or insulator material. Most polymers (including PLA and PCL) are electrically insulator and possess very low electrical conductivities. Therefore, when the MEW process is compared for two different polymers, viscosity of their melt should be considered as the most important property in the interpretation of the results.

All scaffolds are printed in 10 layers with a box structure design. In this design, the fibers in each layer are all parallel to each other and they are perpendicular to fibers in consecutive layers. For example, if the fibers in the first layer are printed in x direction, then, the fibers in the second layer are printed in y direction (perpendicular to fibers in the first layer). The fibers in third layer will be parallel to the fibers in the first layer and perpendicular to those of second layer. In the end, the fibers in the 1st, 3rd, 5th, 7th, and 9th layers are parallel to each other and perpendicular to those in 2nd, 4th, 6th, 8th, and 10th layers. Also, the 2nd, 4th, 6th, 8th, and 10th layers are parallel to each other. The composite PLA/PCL scaffold also has 10 layers with a box structure design. Here, if the first layer starts with PLA, then, 3rd, 5th, 7th, and 9th layers are also made of PLA and the 2nd, 4th, 6th, 8th, and 10th layers are fabricated using PCL. The fibers in layers made of PLA are perpendicular to those in layers printed with PCL and the fibers in layers fabricated using same polymer are parallel to each other.

The MEW process is conducted for all of the samples with appropriate nozzle head velocity and feed rate. In order to visualize the MEW printing process, the process for PLA fibers is provided in Video S1. The process for PLA and PCL filaments is not similar to each other. Compared to PCL fibers, the accuracy of positioning of the PLA fibers on the substrate is less. This can be attributed to the higher viscosity and/or lower electrical conductivity (although both polymers are non-conductive and this is not likely the case) of PLA melt compared to those of PCL melt. Also, because of the higher melting temperature of PLA (150 to 160 °C) compared to that of PCL (approximately 60 °C), the temperature of the heating section of the MEW device is set to different values for each of the two polymers. This temperature for PCL and PLA is equal to 100 °C and 230 °C, respectively, which is much higher than the melting temperature of both polymers. The main reason for using such high temperature is that it helps the fibers in two consecutive layers to fuse and bond to each other better. Values of some of the MEW parameters which are the same for both polymers are mentioned in Table 1.

**Table 1:** Values of several parameters of the MEW process (equal for both PLA and PCL).

Parameter	Feed rate (µl/min)	Collector temperature (°C)	Critical translational speed (mm/s)	Printing speed set in the G-code (mm/s)	Distance between the nozzle head and collector/substrate (mm)
Value	10	60	60	65	5

During the process, electrical charges are induced by the electric field on the melt surface. Since the Kapton sheet (the substrate) is a non-conductive material, after placement of the fibers on the substrate, the induced electrical charges remain on the surface of the fibers and substrate. The fibers in the next layers have the same type of electrical charges that the fibers in the previous layers have and therefore, a repulsive electric force is created between the previously deposited fibers and those that are placing on these fibers. This repulsive force impedes accurate positioning of the fibers in the next layers and deteriorates the desired jetting of the polymer melt. To compensate for the weakened electric field, the applied voltage has to be increased every 2–3 layers by a value between 500 V and 1 kV. The problem of residual electrical charges worsens when the number of the layers increases and eventually, it becomes impossible to fabricate a well-ordered scaffold when the number of layers exceeds 10.

The desired voltage (the voltage that leads to the formation of a stable and favorable jetting mode) for MEW of the first layer of the neat PCL scaffold is around 2–2.5 kV. During the MEW process, as mentioned in the previous paragraph, because of the residual electrical charges, the voltage has to be increased. The desired voltage applied to the metallic nozzle during the final layer (tenth layer) of the PCL scaffold is equal to 4.5–5 kV. For PLA melt, the voltage starts at around 3.5–4 kV and the voltage of the last year is equal to 6–6.5 kV. Applying higher voltages for fabrication of the PLA scaffolds shows that PCL is much more desirable than PLA for MEW process.

Fabrication of the composite PLA/PCL scaffold using the MEW device is a process that places between the fabrication of neat PLA and neat PCL scaffolds in terms of difficulty of the process. Precise MEW of the layers made of PLA is a difficult process while it is easy to deposit the PCL fibers accurately. The problem of residual electrical charges exists here like the previous scaffolds and to resolve it and reach to the desired jetting mode again, applied voltage is increased. It should be noted that since the FDM device used here for the MEW process has just one nozzle head, when fabrication of one layer of the composite scaffold with for example PLA filament is finished, one should stop the FDM device and wait for the temperature of the filament to decrease. When the temperature of the PLA filament reaches to that of the room, it is drawn out of the guide tube and after becoming sure that the print path starting from the gear system to the nozzle head is clear and open, the PCL filament is placed between the gears of the gear system and the MEW process is begun. The same process is repeated when the PCL filament should be replaced with the PLA filament. Therefore, for one composite scaffold to be completely printed, the process of changing and replacing the filaments should be conducted nine times.

In this study, the aim was to investigate the effect of different materials on the properties of the melt electrowritten scaffolds. Since geometrical characteristics like pore size affect the cell-scaffold interactions, the scaffolds should have similar geometry. Therefore, the printing parameters are set to obtain scaffolds with approximately equal pore sizes in the range of 140 µm to 160 µm. It should be noted that difficulties in MEW process of PLA fibers make it nearly impossible to fabricate scaffolds having smaller pore sizes. In the case of fiber diameters, PCL fibers are mostly narrower (with diameters in the range of 5 µm to 15 µm) than PLA fibers (with diameters between 15 and 25 µm). Bakirci et al.^54^ stated that the viscosity of a polymer melt is the parameter affecting the polymer fiber diameter in MEW process. Therefore, it can be concluded that the larger diameter of PLA fibers may be the result of higher viscosity of PLA melt compared to that of PCL melt.

### Morphology and roughness of the scaffolds

**Figure 4:**
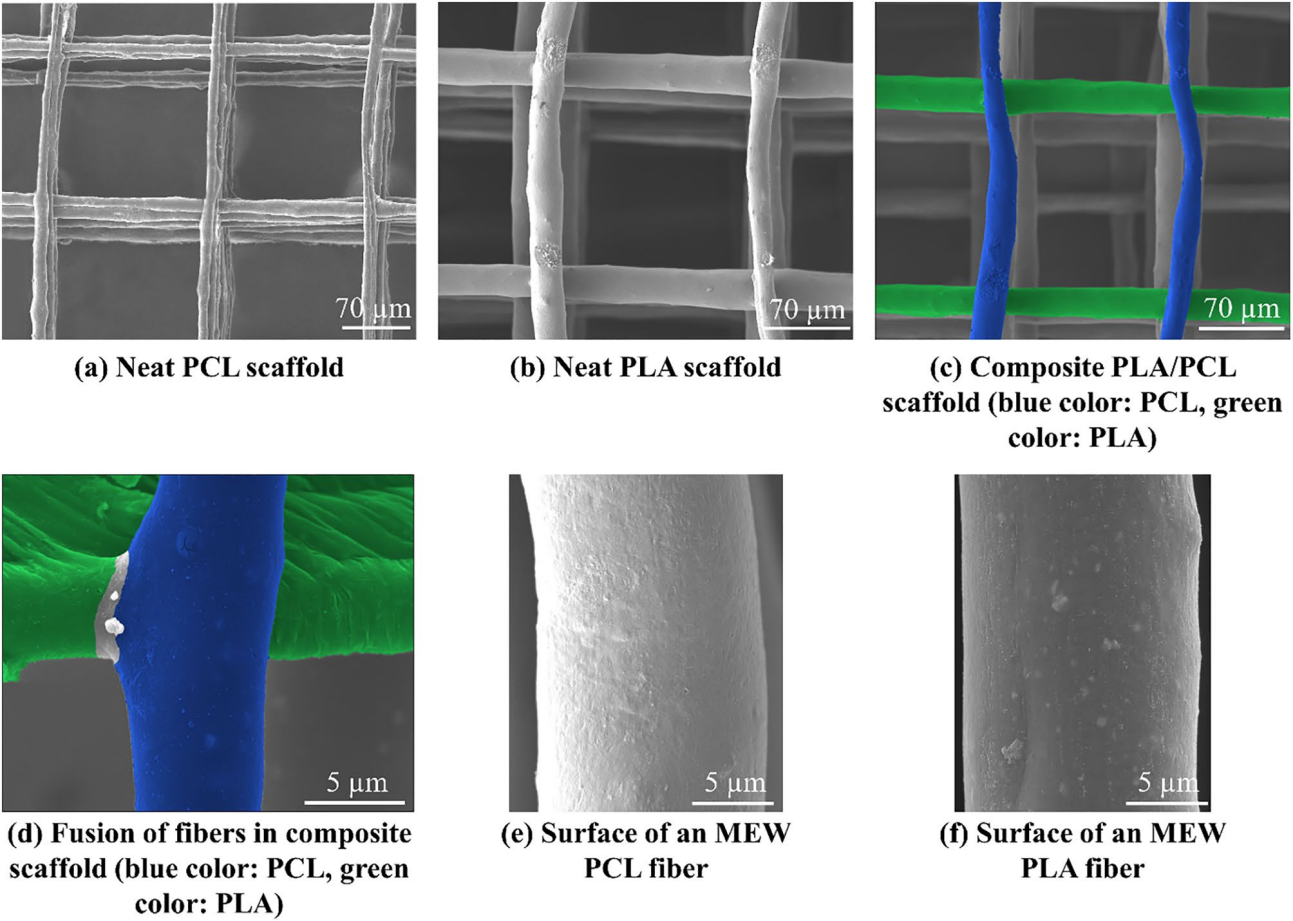
SEM images of the melt elecrowritten scaffolds, (**a–c**) well-ordered box structure of the neat PCL, neat PLA, and composite PLA/PCL scaffolds, respectively, (**d**) bonding and connection of two fibers (the below one is PLA and the upper one is PCL) melt electrowritten in two consecutive layers perpendicularly on each other, and (**e,f**) surface of one of the PCL and PLA fibers, respectively.

Nearly the greatest advantage of MEW over electrospinning is its control on the structure and morphology of the scaffold. This control greatly helps to fabricate scaffolds with more similarity to the structure and geometry of extracellular matrix (ECM) compared to electrospun scaffolds. Therefore, it is of substantial importance to investigate these two features of the melt electrowritten scaffolds (i.e., structure and morphology). In Fig. 4, the SEM images taken in different scales from the scaffolds are shown. It is obvious from SEM images (Fig. 4a–c) that the MEW device is able to fabricate well-ordered scaffolds with perfect box structures (all structures have the same dimensions approximately). Fibers have a uniform cylindrical shape with a nearly constant diameter (for PCL: from 5 to 15 µm, for PLA: from 15 to 25 µm) from the start till the end. Porosity of scaffolds which is one of the most important parameters showing the ability of a scaffold in cell growth and viability is measured using ImageJ software and it is equal to 70% ± 4.5% for all scaffolds.

Connection and bonding between fibers in consecutive layers is another important parameter that can have a significant effect on the mechanical behavior and tensile strength of the scaffold. Strong bonding of two fibers (the upper one is PCL (painted with blue color using MountainsSEM software) and the below one is PLA (painted with green color)) in adjacent layers of the composite PLA/PCL scaffold at the intersection point is observed in Fig. 4d. The color of the area that the two fibers are fused together has not changed to show that there is not any visible barrier in the bonding place. This represents the potential of MEW technique in fabrication of scaffolds with high mechanical standards.

Topography and surface roughness of scaffolds are of substantial importance in their biocompatibility^55^. Faia-Torres et al.^56^ showed that PCL with a surface roughness of 0.93 µm can induce osteogenic differentiation of human mesenchymal stem cells (hMSCs) without any need to special inducer factors. Also, change in surface roughness leads to change in hydrophilicity/hydrophobicity of the surface and this, in turn, affects the cell adhesion onto the surface of scaffolds’ fibers^57^. Surface smoothness and defects are investigated through Fig. 4e,f. There are some defects in the form of bumps, wrinkles, and spots with a different color than that of the surface that are observed on the surface of both fibers and it can be said that the fibers’ surface is not smooth. The dimension of these defects is negligible compared to that of the fibers but their number is relatively significant. These imperfections can be due to detachment of microphase or crystallization induced by the flow^54^.

After qualitatively discussing the topography and surface defects of the fibers, surface roughness of the PCL and PLA fibers is investigated quantitatively using AFM. The results of AFM are demonstrated in Fig. S1a,b for a PCL and PLA fiber, respectively. These results, as explained in the “Materials and methods” section, are in the form of 0.5 µm × 0.5 µm windows in which the height distribution of fiber surface is obtained through scanning the surface using the tip of the atomic force microscope. The maximum height difference between two points in Fig. S1(a) (the PCL fiber) and (b) (the PLA fiber) is equal to 164.5 nm and 80.2 nm, respectively. Furthermore, the RMS value of the surface roughness (i.e., R_q_) is measured using the Gwyddion software and for the PCL and PLA fiber, it is equal to 21.98 nm ± 5.77 nm and 18.73 nm ± 4.90 nm, respectively. According to these values, it is obvious that the PCL fiber has a rougher surface than the PLA fiber. The difference in the values of R_q_ may be due to the different inherent properties of the two polymers and/or different MEW operating parameters (which themselves are affected by the properties of the polymers) used for printing of these polymers. Blum et al.^58^ stated that the surface roughness of melt electrowritten PCL fibers changed from 15 to 5 nm by varying the collector velocity and feeding pressure at the nozzle head. Since they aimed to print fibers with the exact diameter of 5 µm, they had to adjust collector velocity and pressure simultaneously and hence, it was impossible for them to determine which one of the two parameters has the most significant effect on the roughness of the fibers. Values obtained for surface roughness of both PLA and PCL fibers in this study are larger than those achieved in the study of Blum et al.^58^. This can be attributed to the different nozzle head/collector velocity and feed rate/feeding pressure used in the two studies.

### Mechanical behavior of the scaffolds

Mechanical properties of a scaffold such as its ultimate tensile stress (UTS) and Young’s modulus should be as similar as possible to those of the tissue that is going to be substituted with the scaffold. As mentioned in the previous sections, although PCL and PLA both are biocompatible however because of their mechanical properties, they are not perfect for tissue engineering applications. Yao et al.^59^ mentioned that for generation of bone, PCL lacks sufficient elastic modulus and stiffness to support growth of cells and bone regeneration. In addition to this problem, PCL has also other problems like slow degradation rate and lack of bioactivity. To solve these problems, they blended PCL with PLA which possesses a larger strength and Young’s modulus compared to PCL. The composite scaffold obtained by blending PCL and PLA had improved mechanical properties, degradation rate, and bioactivity.

**Figure 5:**
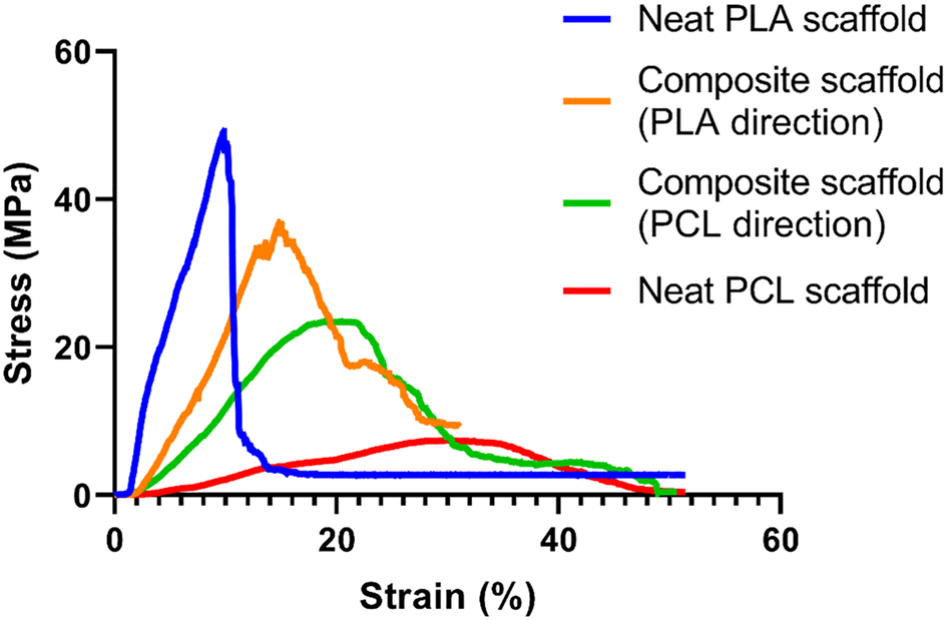
Stress vs. strain curve for 3 scaffolds (the test is conducted in two directions for the composite PLA/PCL scaffold, one is the direction of printing the PLA fibers and the other is the direction of printing the PCL fibers which is perpendicular to the first direction) (number of samples for each measurement = 3) (Statistical analysis with one-way ANOVA shows statistical significance with p-values less than 0.05).

Mechanical behavior of the melt electrowritten scaffolds is investigated in Fig. 5. Using uniaxial tensile test, stress–strain curve is obtained for the three scaffolds fabricated in this study and shown in Fig. 5. Four curves are observed in this figure while three scaffolds have been melt electrowritten. The reason is that the tensile load is applied to two composite PLA/PCL scaffolds. The load is exerted to one of the scaffolds in the direction of melt electrowritten PLA fibers and it is applied to the other scaffold in the orientation that PCL fibers are printed. The stress–strain curves obtained for these two cases are not the same which shows that the composite PLA/PCL scaffold is anisotropic in terms of mechanical properties. Anisotropic mechanical properties are essential in applications like heart tissue engineering. This type of anisotropy is observed in heart valve leaflets^60^. Furthermore, heart tissue demonstrates an inherent anisotropy which helps its muscle to effectively contract and pump the blood^61^. Therefore, since the composite PLA/PCL scaffold fabricated in this study represents an anisotropic mechanical behavior, it can be a promising choice in applications related to heart tissue engineering.

There are three important quantities calculated from the stress–strain curve (Fig. 5). These are UTS, Young’s modulus, and strain to failure which their values can be seen in Table 2, respectively. Based on this table, the neat PLA scaffold has the highest UTS and after this scaffold, the composite PLA/PCL scaffold when tensile load is parallel to PLA fibers (abbreviated as composite scaffold-PLA direction), the composite PLA/PCL scaffold when tensile load is aligned with PCL fibers (abbreviated as composite scaffold-PCL direction), and neat PCL scaffold are placed. In both the neat PLA scaffold and the composite scaffold-PLA direction, the tensile load is applied to PLA fibers but they have different UTS. The reason may be that in the neat PLA scaffold, the PLA fibers are connected in both their lower and upper layers to PLA fibers, while in the composite scaffold-PLA direction, the PLA fibers are bonded to PCL fibers in their upper and lower layers. The bonding and connection help the PLA fibers to tolerate a larger tensile stress until the ultimate point. Since PCL has a lower UTS than PLA, the PLA fibers in the neat PLA scaffold receive a stronger support compared to the PLA fibers in the composite scaffold-PLA direction and this can be the reason that neat PLA scaffold has a larger UTS than composite scaffold-PLA direction. This is exactly the case for the neat PCL scaffold and composite scaffold-PCL direction.

**Table 2:** Ultimate tensile stress, Young’s modulus, and strain to failure of neat PLA, neat PCL, and composite PLA/PCL scaffold (in PLA and PCL directions) (Results in this table are represented as mean ± standard deviation) (number of samples for each measurement = 3) (Statistical analysis with one-way ANOVA shows statistical significance with p-values less than 0.05).

	Neat PLA scaffold	Composite scaffold-PLA direction	Composite scaffold-PCL direction	Neat PCL scaffold
Ultimate tensile stress (MPa)	42.98 ± 5.54	28.87 ± 7.79	22.74 ± 0.89	5.97 ± 1.76
Young’s modulus (MPa)	4.5 ± 0.50	3.13 ± 1.27	1.36 ± 0.15	0.46 ± 0.30
Strain to failure (%)	8.92 ± 3.57	16.50 ± 1.50	17.84 ± 2.76	28.33 ± 7.64

The other mechanical characteristic demonstrated in Table 2 is Young’s modulus. The modulus is defined as the slope of stress–strain curve in the linear elastic region and it is an indication of the stiffness of the material. From Table 2, it can be seen that the neat PLA scaffold which is made of stiff PLA has the largest modulus, while the neat PCL scaffold fabricated from flexible PCL possesses the smallest Young’s modulus. Like the case of UTS, here, the composite scaffold-PLA direction shows a larger modulus than the composite scaffold-PCL direction whereas it has a smaller modulus compared to the neat PLA scaffold. Also, the composite scaffold-PCL direction represents a larger Young’s modulus than the neat PCL scaffold. The results for composite PLA/PCL scaffold are interpreted in a way completely similar to the results of UTS. Here, the presence of PCL fibers bonded to PLA fibers in the transverse direction makes the composite scaffold-PLA direction more flexible compared to the neat PLA scaffold and consequently, decreases its Young’s modulus. For the composite scaffold-PCL direction, the existence of PLA fibers connected and perpendicular to PCL fibers increases the stiffness and elastic modulus of composite scaffold-PCL direction in comparison to neat PCL scaffold.

The strain to failure for the scaffolds is the last mechanical property that its values are mentioned in Table 2. The neat PLA scaffold which is the most brittle scaffold has the smallest strain to failure. Substitution of PLA fibers in neat PLA scaffold with ductile PCL fibers causes the composite scaffold-PLA direction to own a higher strain to failure value than neat PLA scaffold. The composite scaffold-PCL direction has a larger strain to failure than composite scaffold-PLA direction. Also, the value of strain to failure for composite scaffold-PCL direction is smaller than that of the neat PCL scaffold which is because of the presence of brittle PLA fibers instead of ductile PCL fibers in the transverse direction. Finally, the neat PCL scaffold which is fabricated from ductile PCL fibers in both the direction of tensile load and the transverse direction possesses the highest value of strain to failure among the melt electrowritten scaffolds.

### Contact angle and swelling ratio of the scaffolds

Angle and swelling ratio are both an indication of how much the fabricated scaffolds are hydrophilic/hydrophobic. Cell attachment to the surface of a scaffold depends directly to the degree of hydrophilicity/hydrophobicity of the surface. In fact, cells have a great affinity for attaching to hydrophilic surfaces. On the other hand, proteins prefer to adhere to hydrophobic surfaces rather than hydrophilic ones. Considering the role of proteins in cell adhesion, it can be concluded that the optimal cell-scaffold interaction is achieved when there is a balance between the hydrophilicity and hydrophobicity of the surface of the scaffold^55,62^. PCL and PLA, the synthetic polymers used in this study, are both hydrophobic in nature. However, PCL is more hydrophobic than PLA^63^ and this is a great disadvantage for PCL. Therefore, the use of their composite in cell culture and proliferation finds another significant reason that is to add some hydrophilicity to hydrophobic PCL and create the needed hydrophilic/hydrophobic balance. The increase in wettability of the surface by using composite PLA/PCL instead of neat PCL and hence, improving the cell attachment and proliferation have been observed in several studies^46,63^.

The results of contact angle and swelling ratio tests are represented in Table 3. Based on this table, the neat PLA scaffold has the smallest contact angle (90° ± 10°) and largest swelling ratio (21%) which when compared to the larger contact angle (100° ± 5°) and smaller swelling ratio (8%) of the neat PCL scaffold, demonstrates that neat PLA scaffold is more hydrophilic than the neat PCL scaffold. The composite scaffold shows a similar contact angle (100° ± 3°) to the neat PCL scaffold showing that addition of PLA fibers to the structure of the scaffold does not change the contact angle. However, the presence of more hydrophilic PLA fibers increases the water absorbance capability (swelling ratio = 15%) of the composite scaffold and this can positively affect the potential of this scaffold for cell adhesion and proliferation when compared to the neat PCL scaffold.

**Table 3:** Contact angle and swelling ratio of the melt electrowritten scaffolds (number of samples for each measurement = 3) (statistical analysis with one-way ANOVA shows statistical significance with p-values less than 0.001).

	Neat PLA scaffold	Neat PCL scaffold	Composite PLA/PCL scaffold
Contact angle (°)	90 ± 10	100 ± 5	100 ± 3
Swelling ratio (%)	21	8	15

### In vitro biocompatibility and cell attachment

**Figure 6:**
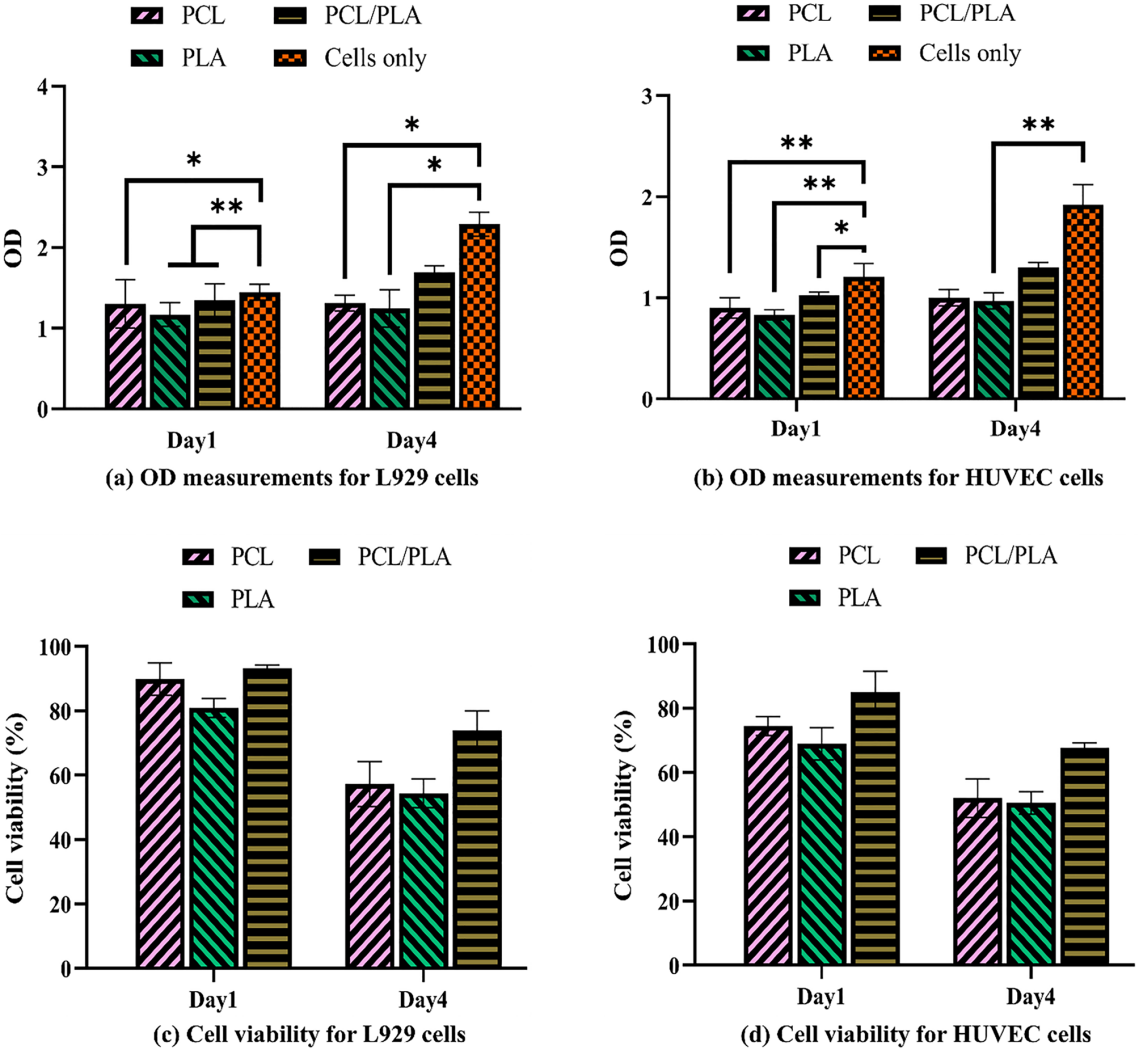
In vitro cytotoxicity and cell viability of the scaffolds, (**a,b**) biocompatibility of the scaffolds for L929 and HUVEC cells obtained by OD measurements, respectively, and (**c,d**) cell viability of the scaffolds for L929 and HUVEC cells, respectively (number of samples for each measurement = 3) (for OD charts: *shows p < 0.05, **demonstrates p < 0.01, and ***represents p < 0.001) (for cell viability charts: statistical significance is shown with negligible p value).

In the current study, the biocompatibility of L929 and HUVEC cells cultured on the polystyrene plate (Control) as well as neat PLA, neat PCL, and composite PLA/PCL scaffolds is assessed using the MTT test. Figure 6a,b show the optical density (OD) measurements for L929 cell-cultured and HUVEC-cultured samples, respectively; according to the results, the OD of all samples rises over time, indicating their biocompatibility. In particular, the composite PLA/PCL sample experiences a significant increase in OD values adjacent to both cell lines after 4 days. This result stems from the synergistic effects of PCL's excellent cell attachment and PLA’s satisfactory mechanical strength, providing support for cell growth, nutrient/gas exchanges, and bioactivity^64–66^ in composite PLA/PCL scaffold.

The viability of L929 and HUVEC cell lines on specimens is provided in Fig. 6c,d, respectively. The neat PLA scaffold has the weakest, and the composite PLA/PCL scaffold has the strongest L929 and HUVEC cell viability on day 1. The neat PCL scaffold has a better cell viability than the neat PLA scaffold on day 1 which is due to its higher hydrophobicity that in turn causes a stronger initial cell adhesion than the neat PLA scaffold^67^; however, approximately the same cell viability is observed for both the neat PLA and neat PCL scaffolds on day 4. This result stems from PLA fibers’ larger mechanical strength and relatively higher stiffness/modulus than PCL fibers, providing better support for cell growth^59,66^. Overall, the proposed melt electrowritten scaffolds provide a better platform for L929 cells proliferation compared to HUVEC cells.

**Figure 7:**
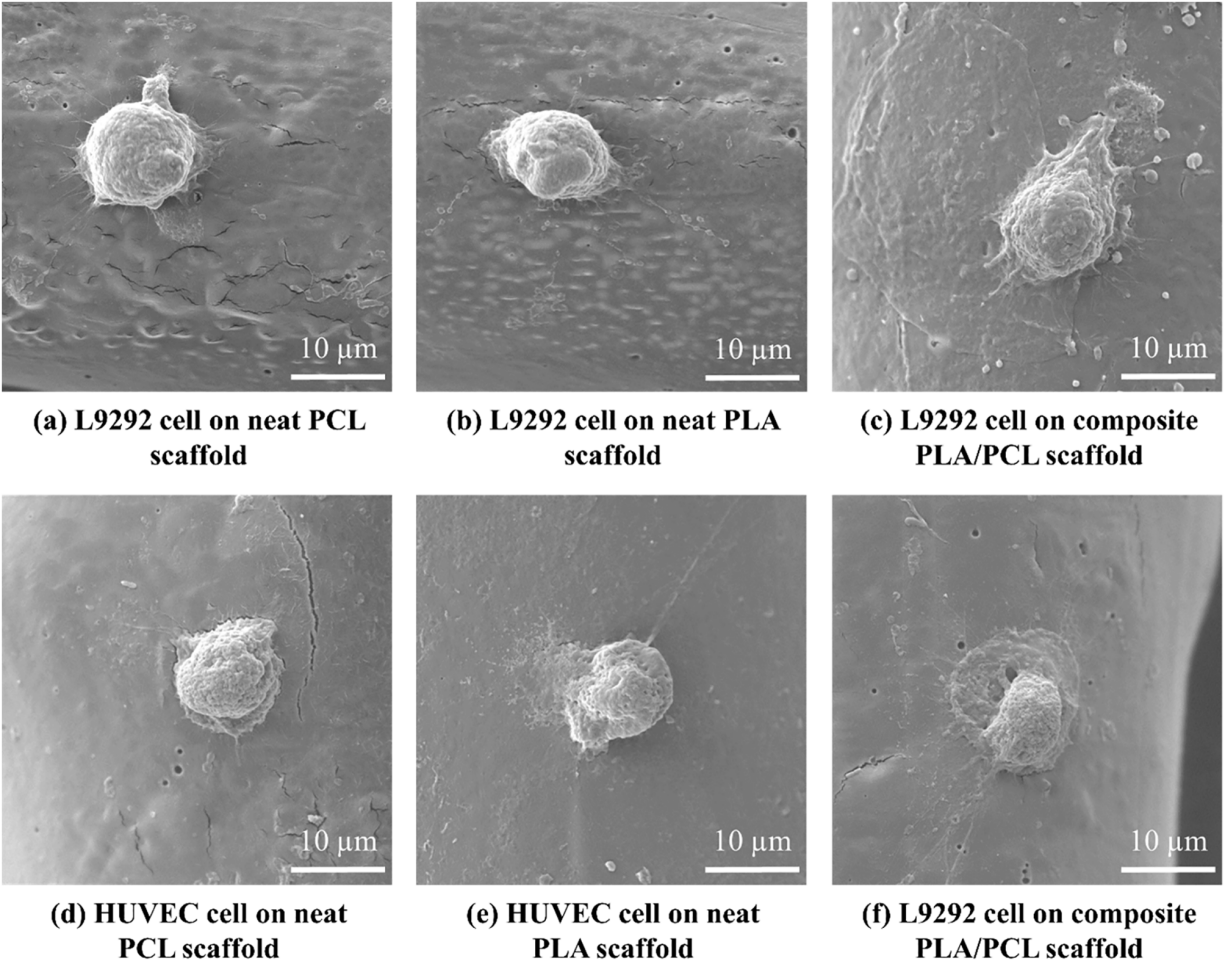
(**a–c**) L929 cells adhesion to the surface of the neat PCL, neat PLA, and composite PLA/PCL scaffolds, respectively, and (**d–f**) attachment of HUVEC cells to the surface of the neat PCL, neat PLA, and composite PLA/PCL scaffolds, respectively.

Concerning the cell attachment results (Fig. 7), L929 and HUVEC cells properly adhere to the scaffolds’ surface; accordingly, L929 cells maintain their spherical shape on the samples, while HUVEC cells slightly spread on the surfaces, especially in the case of the composite PLA/PCL scaffold.

**Figure 8:**
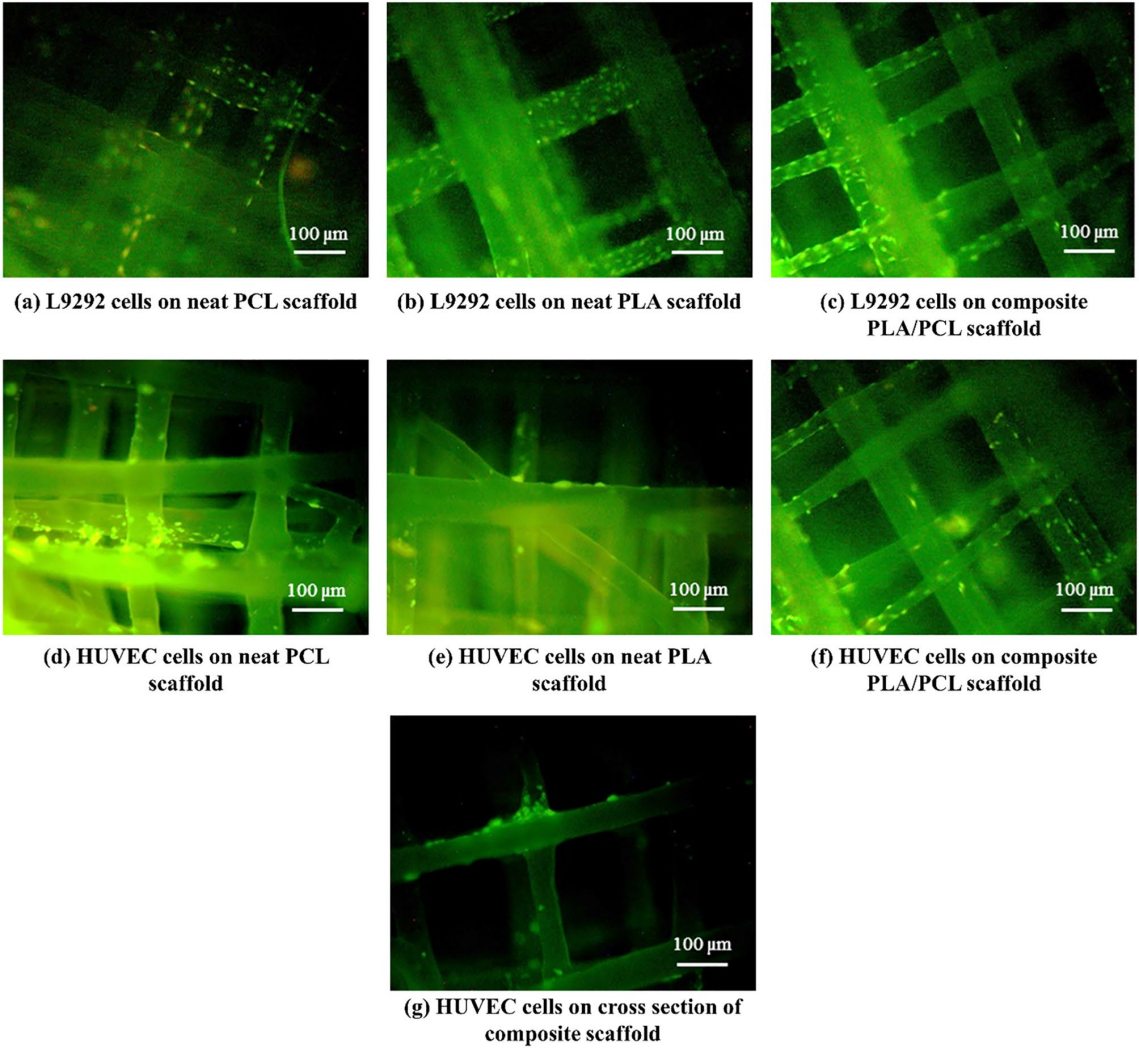
(**a–c**) LIVE/DEAD staining results of L929 cells for the neat PCL, neat PLA, and composite PLA/PCL scaffolds, respectively, (**d–f**) LIVE/DEAD staining results of HUVEC cells for the neat PCL, neat PLA, and composite PLA/PCL scaffolds, respectively, and (**g**) view of HUVEC cells on the crossing fibers of the composite PLA/PCL scaffold showing the large number of alive cells on the intersection.

It can be inferred from LIVE/DEAD staining results that both cell lines proliferate and distribute better on the composite PLA/PCL scaffold compared to two other scaffolds, confirming the MTT results (Fig. 8). An approximately similar morphology is observed for L929 and HUVEC cells on the melt electrowritten scaffolds. Interestingly, cells mostly adhere to the intersection of fibers, where the synergistic effect of PCL and PLA fibers is intense (Fig. 8g).

## Conclusions

In this paper, a recent additive manufacturing technique named melt electrowriting (MEW) was used to fabricate box-structured ten-layer scaffolds. To print scaffolds using this technique, for the first time, a commercial FDM 3D printer with several modifications in its setup was utilized. Three scaffolds were made which were neat PLA, neat PCL, and composite PLA/PCL scaffolds. Composite PLA/PCL scaffold was printed in this study for the first time. The scaffolds were then evaluated in terms of morphology (using SEM), topology and surface roughness (using AFM), mechanical properties (using uniaxial tensile test), hydrophilicity/hydrophobicity (contact angle and swelling tests), and cell growth and viability (by culturing L929 and HUVEC cells on the surface of the scaffolds).

The results demonstrated that although MEW of PLA fibers is more difficult than PCL fibers and the problem of residual electrical charges deteriorates the accurate deposition of fibers, it is possible to fabricate well-ordered scaffolds using PLA filaments. Also, it was shown that there is bonding and connection between PCL and PLA fibers in adjacent layers in composite PLA/PCL scaffold which guarantees the high mechanical strength of the scaffold. AFM results represented that the surface of PCL fibers is rougher than PLA fibers. The difference between the values of the surface roughness of PLA and PCL fibers can be related to the different optimized operating parameters of the MEW process for printing these two materials (like feed rate and nozzle head velocity). According to the results of the uniaxial tensile test, the composite PLA/PCL scaffold represents an anisotropic mechanical behavior with different Young’s modulus in the directions that PLA and PCL fibers are printed. This anisotropy can be used in heart tissue engineering applications. Also, the undesired properties of PLA and PCL (for example high brittleness of PLA or low ultimate tensile stress of PCL) improved in composite PCL/PLA scaffold. PLA is more hydrophilic than PCL and therefore, neat PLA scaffold represented a lower contact angle and larger swelling ratio than neat PCL scaffold. Existence of PLA fibers in composite PLA/PCL scaffold did not change the contact angle, however, it increased the swelling ratio of the scaffold compared to neat PCL scaffold.

The results of MTT test represented that the melt electrowritten scaffolds are all biocompatible. Neat PCL and Composite PLA/PCL scaffolds had the worst and best cell viability values, respectively. Hydrophobicity caused neat PCL scaffold to have a better cell viability than neat PLA scaffold on day 1, while because of the stronger mechanical support provided by neat PLA scaffold, both neat PLA and PCL scaffolds showed a similar cell viability on day 4. SEM images showed that both cell types attach well to the surface of the scaffolds.

Most of the previous studies have used PCL to fabricate melt electrowritten scaffolds. While PCL has several outstanding properties, it is bio-inert, very hydrophobic, and has a low ultimate tensile stress. In this study, by melt electrowriting well-ordered composite PLA/PCL scaffolds, a promising alternative with improved mechanical properties and cell-scaffold interactions was proposed.

## Supplementary Information

Supplementary Information.

Supplementary Video S1.

## Data Availability

The datasets used in the current study is available from the corresponding author on reasonable request.
