# Supplementary material for: Melt electrowriting of PLA, PCL, and composite PLA/PCL scaffolds for tissue engineering application
Source: Sci Rep. 2022 Nov 19;12:19935. doi: 10.1038/s41598-022-24275-6 (PMC9675866; doi:10.1038/s41598-022-24275-6)
Supplement: Supplementary file 1 — Supplementary Information. [file 41598_2022_24275_MOESM1_ESM.docx]

**
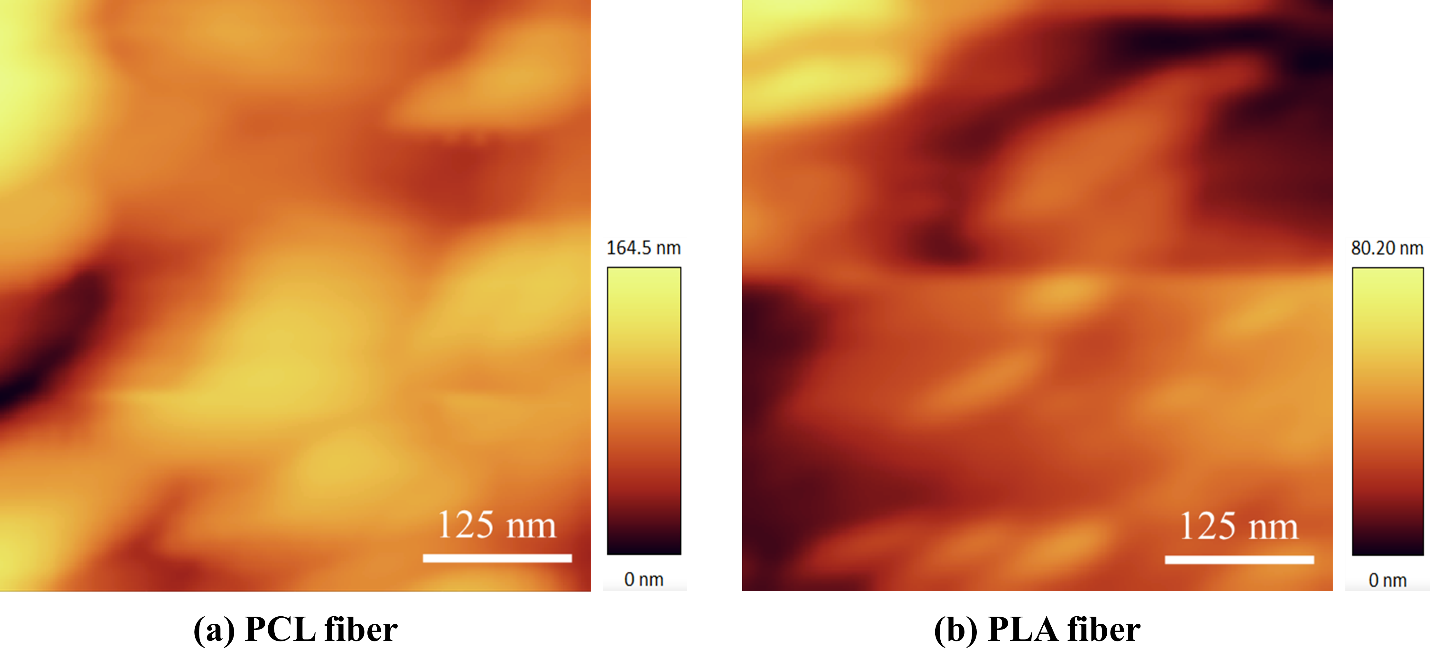
**

Fig. S1 AFM images of (a) a PCL fiber and (b) a PLA fiber

Video S1: Melt electrowriting process for PLA fibers
